# MicroRNA-dependent regulation of Hox gene expression sculpts fine-grain morphological patterns in a *Drosophila* appendage

**DOI:** 10.1242/dev.161133

**Published:** 2018-10-16

**Authors:** Richard Kaschula, Sofia Pinho, Claudio R. Alonso

**Affiliations:** Sussex Neuroscience, School of Life Sciences, University of Sussex, Brighton BN1 9QG, UK

**Keywords:** Drosophila, Hox, Haltere, microRNA, Morphology

## Abstract

Disruptions of normal Hox gene expression can lead to severe morphological defects, revealing a link between the regulation of Hox expression and pattern formation. Here, we explore these links, focusing on the impact of microRNA regulation on the expression of the *Drosophila* Hox gene *Ultrabithorax* (*Ubx*) during haltere development. Through a combination of bioinformatic and transcriptomic analyses, we identify the miR-310/313 cluster (*miR-310C*) as a candidate regulator of *Ubx*. Several experiments confirm this. First, *miR-310C* and Ubx protein show complementary expression patterns in haltere imaginal discs; second, artificial activation of *miR-310C* expression in haltere discs leads to *Ubx*-like phenotypes. Third, expression of a fluorescent reporter bearing *Ubx* 3′UTR sequences is reduced when co-expressed with *miR-310C*. Fourth, deletion of *miR-310C* leads to *Ubx* upregulation and changes the array of mechanosensory sensilla at the base of the haltere. Fifth, an artificial increase of *Ubx* levels within the *miR-310C* expression domain phenocopies the mechanosensory defects observed in *miR-310C* mutants. We propose that *miR-310C*-mediated repression delimits *Ubx* fine-grain expression, contributing to the sculpting of complex morphologies in the *Drosophila* haltere with implications for flight control. Our work reveals a novel role of microRNA regulation in the control of Hox gene expression with impact on morphology.

## Abstract (precis)

**Summary:** A novel role of microRNAs in the control of Hox gene expression with impact on the development of morphology in *Drosophila*, suggesting that microRNAs might play similar roles in other bilaterians.

## INTRODUCTION

The Hox genes encode a family of transcriptional regulators involved in the coordination of the cellular behaviours that lead to the formation of distinct structures along the main body axis of animals. Forty years ago, Ed Lewis (Lewis, 1978) showed that genetic modifications of the *Drosophila* Hox genes could induce radical changes in cellular specification along the body axis, leading to homeotic transformations i.e. the change of one body part into the likeness of another (Bateson, 1894). For example, mutations that abolish expression of the *Drosophila* Hox gene *Ultrabithorax* (*Ubx)* transform the halteres – small metathoracic dorsal appendages essential for normal flight (Fraenkel and Pringle, 1938; Pringle, 1948) – into wings; conversely, ectopic expression of *Ubx* within the developing wing imaginal disc transforms wings into halteres (Cabrera et al., 1985; Lewis, 1963; White and Wilcox, 1985a,b). These experiments, and many others in the following years, made it clear that Hox expression levels must be kept within certain limits for development to proceed normally.

It therefore follows that animals must establish a cellular ‘set-point’ for Hox expression and possess adequate molecular regulatory mechanisms to keep Hox activity levels around such target values (Mallo and Alonso, 2013). Such set points are likely to matter not only during the allocation of the ‘Hox code’ to individual developmental units along the main body axis, but also during the sub-specification of the Hox-dependent programs that lead to the morphogenetic complexity internal to the segment. This latter function directly underlies the generation of fine-grain patterning within segments. Despite this apparent importance, the molecular mechanisms that safeguard Hox expression set points are not well understood. Here, we explore this problem during the formation of the *Drosophila* haltere, focusing on the regulatory roles played by microRNAs (miRNAs).

miRNAs are small non-coding RNAs that are able to repress gene expression by inducing mRNA degradation or blocking translation, thus regulating the net output of target genes (Bartel, 2009; Alonso, 2012). Previous work revealed that the products of the *miR-iab4/8* locus can regulate the expression of posterior Hox genes in the developing haltere: ectopic expression of *miR-iab4* (Ronshaugen et al., 2005) and *miR-iab8* (Stark et al., 2008; Tyler et al., 2008) leads to haltere-to-wing transformations that emerge from differential repression of *Ubx*. However, a precise mutation that abolishes expression of both miRNAs, *miR-iab4* and *miR-iab8*, produces no detectable changes in haltere morphology (Bender, 2008), indicating that these miRNAs play no significant role in the control of normal expression levels of *Ubx* during haltere formation and are thus dispensable for the development of cellular patterns in the haltere. In contrast, a quantitative trait locus study conducted across several *Drosophila melanogaster* populations uncovered a link between the locus encoding *miR-92a* and variation in trichome patterns on the femur of the second leg, in a region termed the ‘naked valley’; yet, multiple genetic tests ruled out any involvement of *Ubx* in the generation of intra-specific variation of naked valley patterns (Arif et al., 2013).

Here, we show that a miRNA system encoded within the *miR-310C* cluster controls *Ubx* expression during haltere development. We also provide evidence that miRNA-dependent fluctuations in *Ubx* expression level determine the arrangement of fine-grain morphological elements known to convey sensory information during flight control. We propose that Hox gene post-transcriptional control via small non-coding RNAs may represent a mechanism that sculpts morphological patterns in developing appendages in other animals too. We also discuss the implications of our findings for the understanding of the molecular programs controlling the morphological specification of appendages.

## RESULTS

### MicroRNAs from the miR-310C locus are predicted to target Ubx and are expressed in the developing haltere disc

**Fig. 1.**
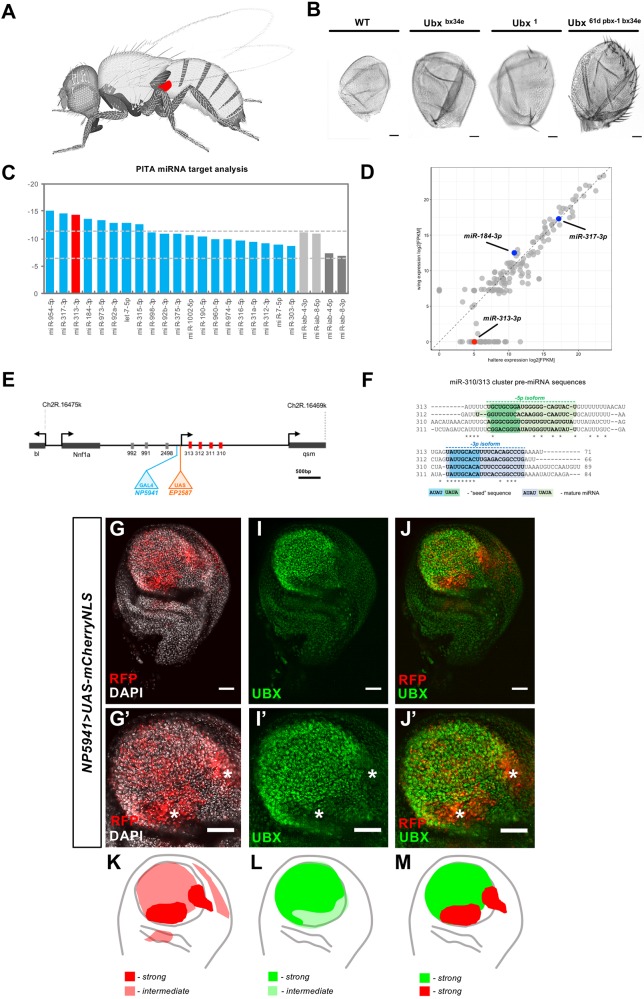
**The *Drosophila* haltere as a system for studying Hox expression control by miRNAs.** (A) Schematics of the adult fly, indicating the position of the haltere (red) on the dorsal region of the posterior thoracic segment (adapted, with permission, from Hartenstein, 1993). (B) Halteres from an allelic series of *Ubx* mutations showing variations in phenotype dependent on the extent of reduction in *Ubx* expression. Scale bars: 10 μm. (C) Plot showing the top 20 candidate miRNAs that target *Ubx* with experimentally tested expression in imaginal discs. Predicted target scores are represented as ΔΔG values (output of PITA algorithm) (the miRNAs *miR-iab-4* and *miR-iab-8* have been previously shown to negatively regulate *Ubx* expression and are hereby included as a point of reference for ΔΔG values, shown in grey). (D) Scatterplot showing normalised RNA sequencing reads of microRNAs within the developing haltere and wing imaginal discs. Highlighted are *miR-313*, which is found solely within haltere-read data, compared with *miR-184-3p*, which is detected solely within wing discs, and *miR-317-3p*, which is detected in both tissues. (E) Genomic map of the *miR-310-313* (*miR-310C*) cluster. Two P-element insertion sites are shown: *miR-310C^EP2587^* (UAS::*miR-310C*) carrying multiple UAS promoter sequences; and *miR-310C^NP5941^*(miR310C::GAL4) containing a GAL4 coding sequence. (F) Alignment of the *miR-310C* pre-miRNA sequences. 5’ and 3’ mature miRNAs are coloured green and blue, respectively. Lighter shaded areas represent the mature miRNA sequence, darker shaded areas highlight the putative ‘seed’ region of each mature miRNA. (G,G′) *miR-310C^NP5941^* (*miR310C::GAL4*) was used to drive expression of a mCherry reporter (*UAS-mCherryNLS*) in the haltere imaginal disc co-stained with the nuclear marker DAPI. Areas of high *miR-310C* expression are marked by asterisks. (I,I′) Haltere disc stained for Ubx protein expression. Variation in signal intensity can be observed within different regions of pouch, marked by asterisks. (J,J′) Expression of *miR-310C* and *Ubx* within the haltere imaginal disc. Areas of high *miR-310C* expression overlap with low *Ubx* signal intensity (asterisks). Scale bars: 30 μm for G-J′. (K-M) Schematics of a haltere imaginal disc showing regions of high and intermediate levels of *miR-310C* (K) and *Ubx* (L) expression, and strong expression of both (M).

To explore the impact of miRNA regulation on the expression of Hox genes, we decided to focus on those miRNAs able to repress *Ubx* during the formation of the haltere. The haltere (Fig. 1A) is a good system with which to track miRNA-Ubx interactions given that fluctuations in *Ubx* expression level lead to tractable changes in haltere morphology (Fig. 1B), facilitating the evaluation of miRNA-Ubx functional links by means of detailed morphological analysis.

To determine which miRNAs might influence *Ubx* expression during haltere development, we first used an established bioinformatics platform (PITA) (Kertesz et al., 2007; Patraquim et al., 2011), which is able to detect miRNA-specific target sequences present within a specific 3′UTR sequence. PITA analysis of *Ubx* 3′UTR sequences highlights several miRNAs as potential regulators of *Ubx* (Fig. 1C), of which the top five candidates were *miR-954-5p*, *miR-317-3p*, *miR-313-3p*, *miR-184-3p* and *miR-973-5p* (Fig. 1C).

In order to identify miRNAs that might exert their effects on Ubx within the developing haltere, we conducted a transcriptomic analysis that extracted the full miRNA catalogue of the haltere and wing imaginal discs (R.K., A.D. Bomtorin, Z. L. P. Simoes and C.R.A., unpublished). This experiment revealed that *miR-313-3p* was expressed in the haltere at particularly high levels. In contrast, the other two miRNAs showing the highest PITA scores were either absent from both tissues (*miR-954-5p* and*, miR-973-5p*) or expressed in both wing and haltere (*miR-317-3p* and *miR-184-3p*) (Fig. 1D). Taking into account both bioinformatic and transcriptomic information, we decided to focus our work on *miR-313*.

Inspection of the genomic locus encoding *miR-313* revealed that this miRNA is part of a highly compact 1 kb gene complex (i.e. the *miR-310C* cluster, *miR-310C*) (Fig. 1E) that includes a small group of closely related miRNAs (*miR-310*, *miR-311* and *miR-312*) bearing similar core seed-sequences (Fig. 1F), suggesting equivalent mRNA target preferences. Despite this, each miR-310C miRNA might target Ubx with varying ability, owing to differences in mature miRNA sequence composition outside the core-seed sequence. To examine the relationship between the expression patterns of *miR-313* (and sibling miRNAs within *miR-310C*) and *Ubx* within the developing haltere, we used a Gal4-transcriptional reporter inserted in the proximity of the transcriptional start site of *miR-310C* and tracked its expression using a UAS-mCherry reporter construct. Expression analysis in the developing haltere, notably within the pouch region, shows an inverse correlation between the levels of Ubx protein and areas of *miR-310C* expression (Fig. 1G-J′): weakest expression of *Ubx* is detected in regions with highest signal of *miR-310C*; and, reciprocally, high levels of Ubx are detected in areas with low *miR-310C* expression (Fig. 1K-M). This reciprocity seems to be limited to the pouch region where the highest levels of miR-310C are associated with decreased Ubx protein level. These observations are consistent with the hypothesis that Ubx protein expression is regulated via *miR-310C* repression.

### Increased expression of *miR-310C* represses endogenous *Ubx* expression and leads to a Ubx-like haltere-to-wing homeotic transformation

**Fig. 2.**
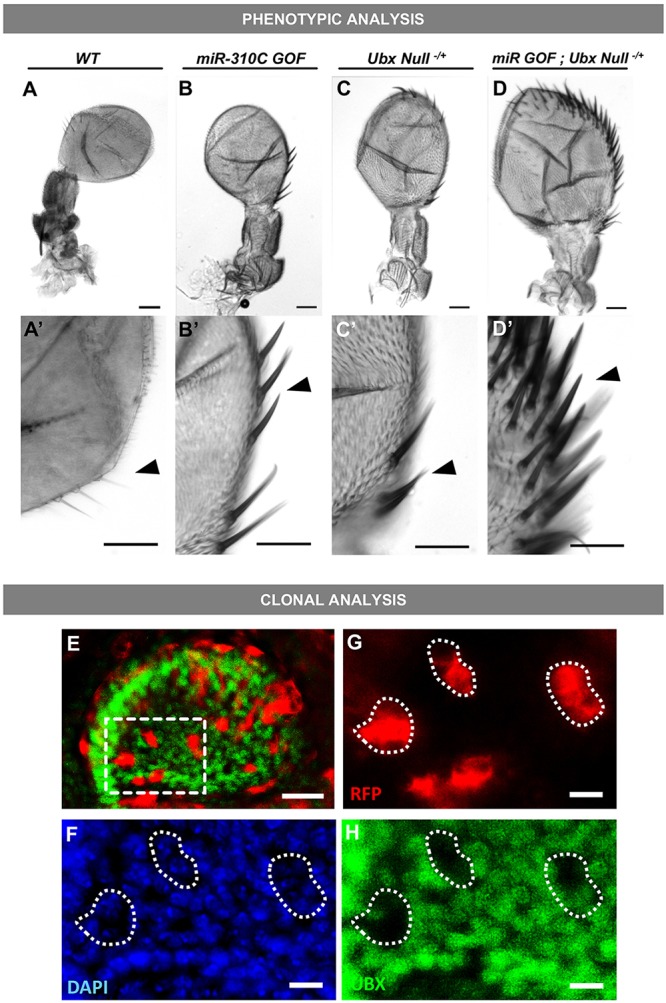
**Phenotypic and clonal analyses of halteres with normal or modified *miR-310C* and *Ubx* expression patterns.** (A-D) Increased expression of *miR-310C* miRNAs using the *miR-310C^EP2587^* (UAS::*miR-310C*) insertion leads to homeotic transformations. (A,A′) Large sensory bristles are normally absent (arrowhead) from wild-type (WT) halteres. (B,B′) miRNA gain-of-function obtained by *nubbin::GAL4*-driven expression of *miR-310C^EP2587^* (*nubbin::GAL4>UAS::miR310C*) leads to the appearance of large ectopic sensory bristles (arrowhead). (C,C′) Haltere from a *Ubx*-null heterozygote (*w;; abx^1^ bx^3^ pbx^1^/+*) shows the appearance of ectopic bristles (arrowhead), representing partial haltere-to-wing homeotic transformations. (D,D′) *miR-310C* gain-of-function in the same *Ubx*-null genetic background (*w; nubbin::GAL4/UAS::miR310C; abx^1^ bx^3^ pbx^1^/+*) leads to a severe haltere-to-wing transformation (arrowhead). (E) Induction of clonal cells in haltere imaginal disc using the FRT ‘Flp-Out’ technique. Cells overexpressing *miR-310C* are also marked by *myrRFP* (*hsFLP^1^;UAS-miR-310C/Actin.STOP.GAL4;UAS-myrRFP)*. (F-H) The same field of clonal cells co-stained for DAPI (F), *miR-310C* (G) and for Ubx protein expression (H). Decreased expression of Ubx is seen in clonal cells (white dashed circles). Scale bars: 40 μm for A-D,E; 25 μm for A′-D′; 10 µm for G-H.

The possibility that *miR-310C* could repress *Ubx* within the context of haltere development leads to the prediction that forced expression of *miR-310C* must lead to a decrease in *Ubx* expression and, depending on the magnitude of such effect, to a potential *Ubx*-like transformation in the haltere. To test these predictions, we expressed the products of *miR-310C* within the developing haltere using a *UAS-miR-310C* transgene and examined the resulting effects on haltere morphology. The results of this experiment (Fig. 2A-B) support the existence of a functional interaction between *miR-310C* and *Ubx*. Overexpression of *miR-310C* leads to the development of ectopic sensory bristles along the margin of the capitellum of the haltere (Fig. 2B-B′) that are very distinct from the normal smaller, lightly pigmented trichomes commonly found in the haltere (Fig. 2A) and constitute a landmark phenotype of haltere-to-wing homeotic transformations observed in *Ubx* mutants (Fig. 1B) (Bender et al., 1983). However, these haltere phenotypes could potentially emerge from effects of *miR-310C* on other genes involved in haltere development. To further delimit the links between *miR-310C* activity and *Ubx*, we applied a genetic approach; this was based on the prediction that increased expression of *miR-310C* in the haltere of an individual heterozygous for a null *Ubx* mutation (i.e. *Ubx^−/+^*) should lead to a more pronounced reduction of *Ubx* expression, causing an even stronger *Ubx* loss-of-function phenotype in the haltere. In line with this prediction, we observed that individuals expressing *miR-310C* within the haltere of the heterozygous *Ubx* mutants *abx^1^*, *bx^3^* and *pbx^1^* (*Ubx^abx, bx, pbx^*) produce halteres with substantial haltere-to-wing transformations (Lewis, 1982) (Fig. 2D-D′). This provides further support for a repressive role of *miR-310C* on *Ubx*. Furthermore, clonal activation of *miR-310C* within the *Ubx* expression domain in the haltere leads to pronounced reductions of *Ubx* expression within the clone, further confirming the repressive activity of *miR-310C* on *Ubx* expression within the developing haltere (Fig. 2E-H).

### Evidence for direct action of miR-310C regulation on Ubx mRNAs

**Fig. 3.**
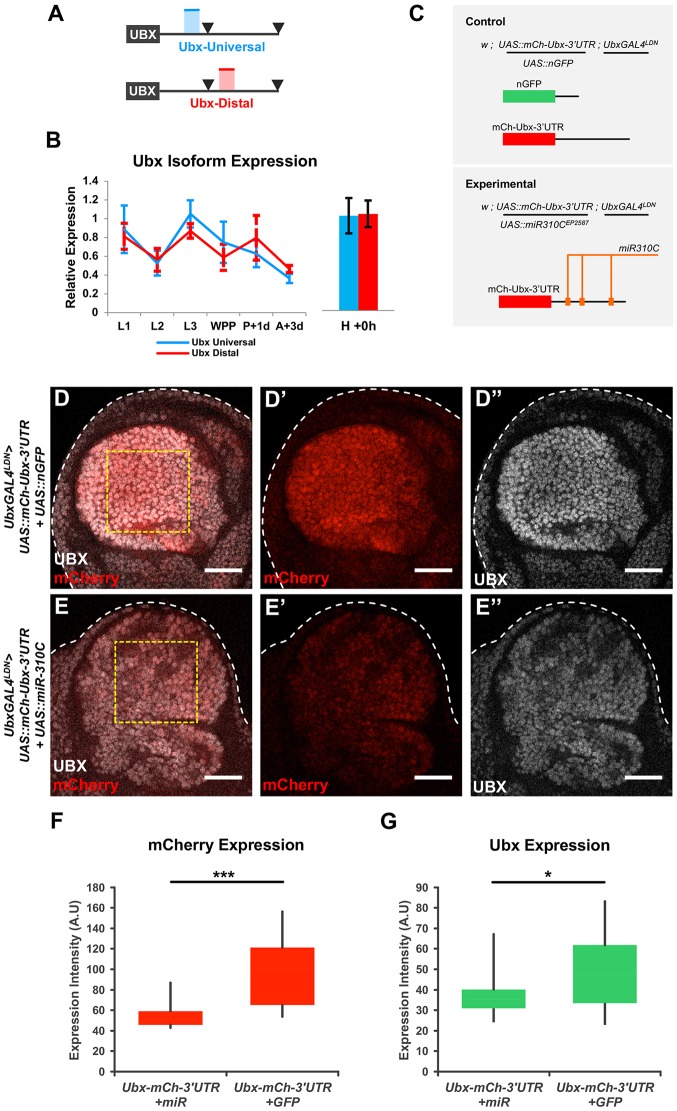
**Developmental analysis of Ubx 3′UTR reporter expression.** (A,B) Analysis of *Ubx* 3′UTR isoform expression during post-embryonic development. (A) PCR primers were specifically designed to detect all *Ubx* transcripts (Ubx Universal, shown in blue) or *Ubx* transcripts containing extended 3′UTRs (Ubx Distal, red). Polyadenylation sites are indicated with arrowheads. (B) Relative expression levels of Ubx 3′UTR isoforms (Ubx Universal versus Ubx Distal) during post-embryonic development (L1, L2, L3, WPP, P+1d and A+3d) and within haltere imaginal discs (H+0 h), plotted in blue and red, respectively. Three technical replicates were averaged for each biological sample. Error bars represent s.d. between biological replicates. (C) To test the ability of *miR-310C* miRNAs to target the Ubx-3′UTR, we co-expressed a *UAS::mCherry-Ubx-3′UTR* reporter construct (mCherry linked to an extended Ubx 3′UTR sequence lacking the first polyadenylation site) alongside a *UAS::miR310C^EP2587^* insertion, and monitored mCherry and Ubx expression levels (see ‘Experimental’). Additionally, *UAS::mCherry-Ubx-3′UTR* was co-expressed alongside *UAS::nGFP*, to control for GAL4 titration between the two experimental genotypes (see ‘Control’). (D-D″) Haltere imaginal discs expressing the *UAS::mCh-Ubx-3′UTR* reporter construct driven by a *Ubx::GAL4^LDN^* enhancer trap co-expressed alongside *UAS::nGFP (w; UAS::nGFP/+; Ubx::GAL4^LDN^/UAS::mCh-Ubx-3′UTR)* (GFP signal not shown). (E-E″) Haltere imaginal disc expressing a *UAS::mCh-Ubx-3′UTR* reporter construct and the *UAS::miR310C^EP2587^*, driven by the *Ubx::GAL4^LDN^* enhancer trap (*w; UAS::miR310C^EP2587^/+; Ubx::GAL4^LDN^/UASmCherry-Ubx 3′UTR*). For both genotypes, mCherry expression was detected using RFP antibody. (F) Quantification of mCherry levels across genotypes. Mean expression intensity was quantified within a defined region of interest within the pouch region of the haltere imaginal disc (D and E, yellow box). (G) Quantification of Ubx protein levels across genotypes using the same region of interest as for mCherry quantification. Statistical significance was calculated using Student's *t*-test (***P<0.001, *P<0.5). Experimental (*Ubx-mCherry-3′UTR+miR-310C*), *n*=20; Control (*Ubx-mCherry-3′UTR+GFP*), *n*=18. The size of the boxes represents the range of values that fit between the 25th and 75th percentiles of the distribution; the bottom and top whiskers represent the minimum and maximum values of the distribution, respectively. Scale bar: 30 μm.

The expression data and genetic interaction experiments presented above strongly indicate that *miR-310C* can reduce the expression of *Ubx* within the developing haltere. Yet these observations do not allow us to establish whether the effects of miRNA regulation on *Ubx* expression are direct or indirect. One way to tackle this issue is the use of a *Ubx* 3′UTR reporter bearing the normal *Ubx* 3′UTR tail linked to a fluorescent reporter (mCherry). However, previous work in our laboratory has revealed that during embryogenesis several Hox genes – including *Ubx* – can produce mRNAs with distinct 3′UTR sequences as a result of alternative polyadenylation (Alonso, 2012; Thomsen et al., 2010). It was therefore important to first establish the type of 3′UTR produced by *Ubx* during post-embryonic development. To achieve this, we quantified the expression levels of short 3′UTR isoforms (universal) as well as long 3′UTR isoforms (distal) during larval, pupal and adult stages (Fig. 3A,B). These experiments revealed that *Ubx* produces mRNA isoforms with long 3′UTR across these developmental stages (Fig. 3B). Based on this information, we co-expressed a reporter bearing *Ubx* long 3′UTR sequences [UAS-mCherry-Ubx 3′UTR long, mCh-Ubx-3′UTR (Thomsen et al., 2010)] and the miR-310 products (UAS-*miR-310C*) within the *Ubx* transcriptional domain in the developing haltere disc (Fig. 3C-E). Being aware that the simple addition of an extra UAS component may titrate away some of the Gal-4 transcriptional capacity – leading to an artefactual decrease in reporter expression – we used as a control a genetic background in which the *Ubx-Gal4* was combined with the mCherry Ubx 3′UTR reporter together with a UAS-nGFP transgene (Fig. 3C). Quantification of expression levels of the mCherry Ubx 3′UTR reporter in the absence (Fig. 3D) and presence (Fig. 3E) of *miR-310C* activity showed clear statistically significant differences, indicating that the miRNA system functionally interacts with sequences in the 3′UTR of *Ubx* (most likely via direct contacts), decreasing reporter expression within the haltere imaginal disc (Fig. 3F). Furthermore, expression levels of the endogenous Ubx protein in the same specimens paralleled the effects observed on the 3′UTR reporter, confirming the capacity of the miRNA system to decrease Ubx expression within the physiological context of the developing haltere (Fig. 3G).

### Deletion of miR-310C leads to increased Ubx expression and changes in haltere morphology

**Fig. 4.**
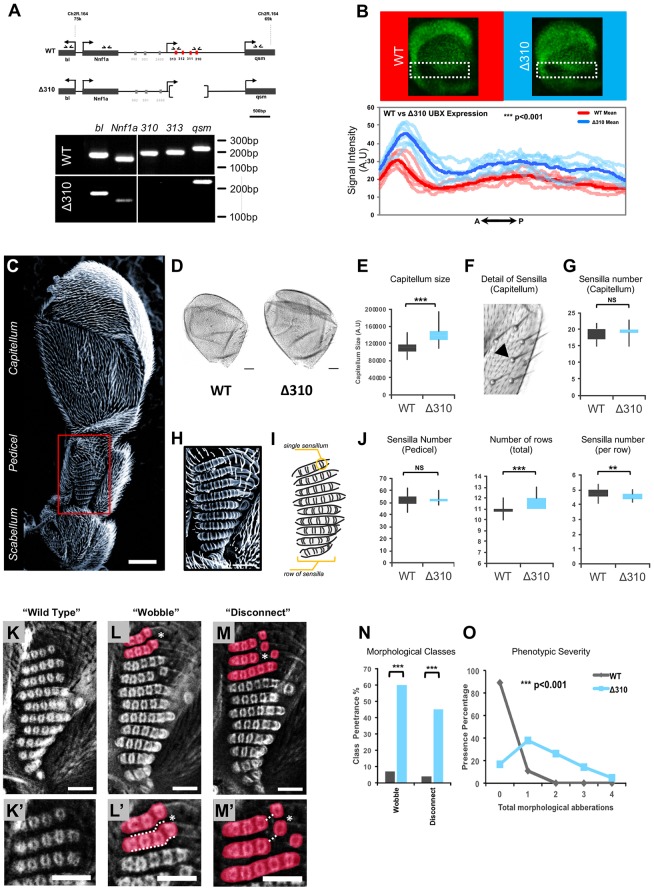
**Mutation of *miR-310C* leads to *Ubx* upregulation and morphological changes in the haltere.** (A) Genomic map of the *miR-310C* cluster showing the region deleted in the Δ310 strain. Genomic PCR confirms that the Δ310 deletion does not affect neighbouring genes (small horizontal arrows mark primer positions for each gene). (B) Plot showing profile of *Ubx* expression intensity across the anterior-posterior axis of the haltere imaginal disc in wild-type and *w;Δ310/Δ310* (Δ310) genotypes. Average intensities were calculated for both wild-type (red) and Δ310 (blue) genotypes across regions within the box; expression profiles from individual samples are shown in light red (wild type) and light-blue (Δ310). Statistical significance was determined using Wilcoxon rank-sum test, ***P<0.001. (C) Scanning electron microscopy image of the haltere showing its three distinctive regions: the scabellum, pedicel and capitellum. (D) Comparison of wild-type and Δ310 halteres. Δ310 halteres have visibly larger capitella. (E) The distribution of capitella size was determined in samples of wild-type and Δ310 genotypes. Comparison of genotypes shows a significant increase in the absence of *miR-310C*. (F) Sensilla are commonly found within the capitellum and display a stereotypical morphology and arrangement (arrowhead). (G) The number of sensilla in the capitella of wild-type and Δ310 halteres was quantified. No significant difference in distribution was detected across the genotypes (*n*=46 for wild type and 42 for Δ310). Statistical significance tested using Student's *t*-test (two-tailed). (H) Scanning electron microscopy image of the dorsal pedicel sensory field of the haltere. (I) Schematics of the sensory fields of the dorsal pedicel; the sensilla are organised in rows. (J) Analysis of sensory cell numbers in the dorsal pedicel of wild-type and Δ310 (*w; Δ310/Δ310*) halteres. Total number of sensilla and number of rows were quantified. The average number of sensilla per row was calculated by dividing total sensilla number by the number of rows. Data shown are a distribution of quantifications for each genotype. Statistical significance was determined using Student's *t*-test (two-tailed). (*n*=30 for wild type and 19 for Δ310). (K,K′) Sensory field of a wild-type dorsal pedicel. (L-M′) Examples of ‘wobble’ and ‘disconnect’ morphological classes. (N) Phenotypic penetrance was calculated for each morphological class comparing wild-type and Δ310 (*w; Δ310/Δ310*) genotypes. Statistical significance was calculated using Fishers exact test, ***P<0.001. (O) Phenotypic severity for wild-type and Δ310 genotypes was determined by quantifying the total number of morphological aberrations per sample. Differences in distributions for each genotype were then tested for statistical significance using Wilcoxon rank-sum test. For analysis of phenotype penetrance and severity *n*=46 for wild type and 42 for Δ310. ***P<0.001, **P<0.01, *P<0.05. In E,G,J, the size of the boxes represents the range of values that fit between the 25th and 75th percentiles of the distribution; the bottom and top whiskers represent the minimum and maximum values of the distribution, respectively. Scale bars: 25 µm in C,D; 10 µm in H,K-M.

A direct way to demonstrate the regulatory effects of *miR-310C* expression on Ubx expression is by mutating or deleting the miRNA gene: elimination of a functional, physiologically relevant repressor of *Ubx* is expected to increase Ubx protein expression in those subsets of haltere cells where the two genes are co-expressed. To test this prediction, we employed a deficiency that removes the entire *miR-310C* locus (i.e. Δ310) (Tang et al., 2010) (Fig. 4A). Molecular analysis of the *miR-310C* locus confirmed that miRNA encoding sequences are absent in this stock (Fig. 4A, lower panel).

Quantitative expression analysis of Ubx protein expression in the pouch region of wild-type and Δ310 mutant haltere imaginal discs revealed a statistically significant increase in the expression of Ubx in the absence of the miRNA system, in line with the model that a functional interaction of *miR-310C* and Ubx products takes place during haltere development (Fig. 4B). Of note is the observation that the normal expression domain of Ubx seen in wild-type haltere discs is maintained in Δ310 mutants, suggesting that these microRNAs exhibit no major effect on the Ubx spatial domain.

We then asked whether miRNA-dependent repression of Ubx had any impact on haltere formation. Previous work on *miR-310C* reported that there were no obvious phenotypic changes in morphology resulting from miRNA deletion (Tang et al., 2010; Tsurudome et al., 2010). Therefore, we considered that should miRNA removal lead to any defects at all in haltere morphology, these were likely to be very subtle. To maximise our sensitivity to detect subtle haltere morphological changes, we combined scanning electron microscopy (SEM) and light microscopy analyses in order to examine haltere morphology in the highest detail.

The haltere can be divided into three anatomically distinct regions: (1) the capitellum, a conspicuous, distally arranged balloon-like compartment; (2) the scabellum, the base of the appendage and the functional point of attachment to the body wall; and (3) the pedicel, which includes the interconnecting tissue that links the capitellum to the scabellum (Fig. 4C). Each compartment is equipped with a number of mechanosensory cells that are disposed in various sensory fields throughout the haltere (Cole and Palka, 1982). Within each compartment of the haltere, sensory elements are formed with distinctive spatial arrangements (Fig. 4C).

Quantification of capitellum size in both normal and miRNA mutant flies revealed a statistically significant difference in the dimensions of this area of the haltere: miRNA-deficient flies produced capitella that were consistently larger than those present in wild-type flies (Fig. 4D-E). Analyses of fine-grain morphological elements within the capitellum, such as mechanosensory sensilla, showed no apparent differences among the genotypes (Fig. 4F-G), suggesting that miRNA-dependent effects on haltere morphology may be related primarily with size-control processes rather than affecting patterning.

The pedicel is almost entirely covered with an array of mechanosensory elements that collect important sensory information for flight control (Dickinson, 1999). Thus, it was of interest to establish whether these physiologically relevant elements were altered by changes in miRNA regulation. In addition, the regularity of the array of mechanosensory sensilla on the pedicel (Fig. 4H-I) represented an excellent ‘read out’ for evaluating the impact of miRNA control on morphological traits. In the normal haltere, sensilla are organised in rows, each one bearing several individual sensillum units (Cole and Palka, 1982; Pringle, 1948). Quantification of the distribution of total sensory cell numbers in the dorsal pedicel of wild-type and Δ310 mutant flies showed that the total number of sensilla per pedicel was not affected by the miRNA mutation (Fig. 4J, left). However, high detail analysis revealed that both the numbers of rows and the number of sensilla per row within the sensory field were significantly different when normal and Δ310 genotypes were compared. Flies lacking the miRNA system had an increase in sensilla rows (Fig. 4J, middle) and a decrease in the number of sensilla included in each row (Fig. 4J, right). Thus, flies lacking normal miRNA regulation produced the normal number of sensilla but in the wrong arrangement. Further characterisation of pedicel patterning showed new distinct features in the patterning of pedicel sensilla in miRNA mutants. Unlike normal halteres, where individual sensilla are regularly positioned next to one another (Fig. 4K-K′), Δ310 mutants frequently show one or several sensilla either out of frame (i.e. ‘wobble’) (Fig. 4L-L′) or spatially separated (i.e. ‘disconnect’) (Fig. 4M-M′) from their respective rows. Although in limited instances we were also able to see some morphological alterations on the ventral side of the pedicel (i.e. a wobble-like phenotype), changes in this region were of much lower magnitude and appeared at a very reduced frequency that did not attain statistical significance. We also observed weak effects on the patterning of the scabellum. But none of these changes had the strength and penetrance of those on the dorsal pedicel. Indeed, penetrance analyses of both phenotypic defects found on the dorsal pedicel (wobble and disconnect) showed differences in the order of tenfold (Fig. 4N), with wobble phenotypes observed in almost 60% of cases in mutants compared with only 7% of wild-type specimens and the disconnect phenotypes present in 45% of samples (in contrast to fewer than 4% of this type of anomaly present in normal halters). Grouping all distortions in sensilla patterning and orientation observed in mutant and wild-type samples reveals that overall phenotypic severity is markedly different between miRNA-deficient and normal flies (Fig. 4O). Altogether, these findings suggest that miRNA regulation is involved in both appendage growth control and the production of ‘fine-grain’ patterning of morphological elements with established physiological relevance in the transduction of sensory information (Cole and Palka, 1982).

### Upregulation of Hox expression within the miR-310C expression domain phenocopies haltere patterning defects observed in miRNA mutants

miRNAs are gene regulatory agents and, accordingly, are not expected to exert direct effects on the cellular processes underlying sensilla patterning in the haltere. Instead, miRNA roles may affect the biology of the cell by means of regulatory roles on miRNA-target genes (Alonso, 2012; Bartel, 2009; Bushati and Cohen, 2007; Pasquinelli, 2012). Exploring candidate target genes whose regulation may be distorted in the haltere, we considered the Hox gene system, and in particular the *Ubx* gene. As shown above (Figs 3 and 4) *Ubx* is a genuine target of the *miR-310C* within the developing haltere and, as such, represents a potential genetic link between the activity of *miR-310C* and the processes that control haltere formation. In this scenario, an artificial increase in Ubx expression within those cells normally expressing *miR310C* products should in some way be able to recapitulate or phenocopy the defects in haltere growth and patterning observed in miRNA mutants. We tested this idea by overexpressing *Ubx* within the developing haltere using the *miR-310C* transcriptional driver and analysed the size and patterning of the resulting halteres.

Sustained overexpression of *Ubx*, either from first (L1) or second (L2) larval instars, was insufficient to cause an increase in haltere size such as the one observed in miRNA mutants (Fig. S1). Furthermore, the combination of the miRNA mutation – expected to lead to an increase in *Ubx* expression– with a null *Ubx* allele *(abx^1^ bx^3^ pbx^1^)* in heterozygosity – predicted to restore, at least to some degree, normal levels of *Ubx* expression – did not lead to any detectable improvement in the abnormal haltere size observed in miRNA mutants; in fact, this new genetic configuration led to an increase in haltere size (Fig. S1). These results suggest that *miR310C* exerts its roles in haltere size control in a *Ubx*-independent manner.

**Fig. 5.**
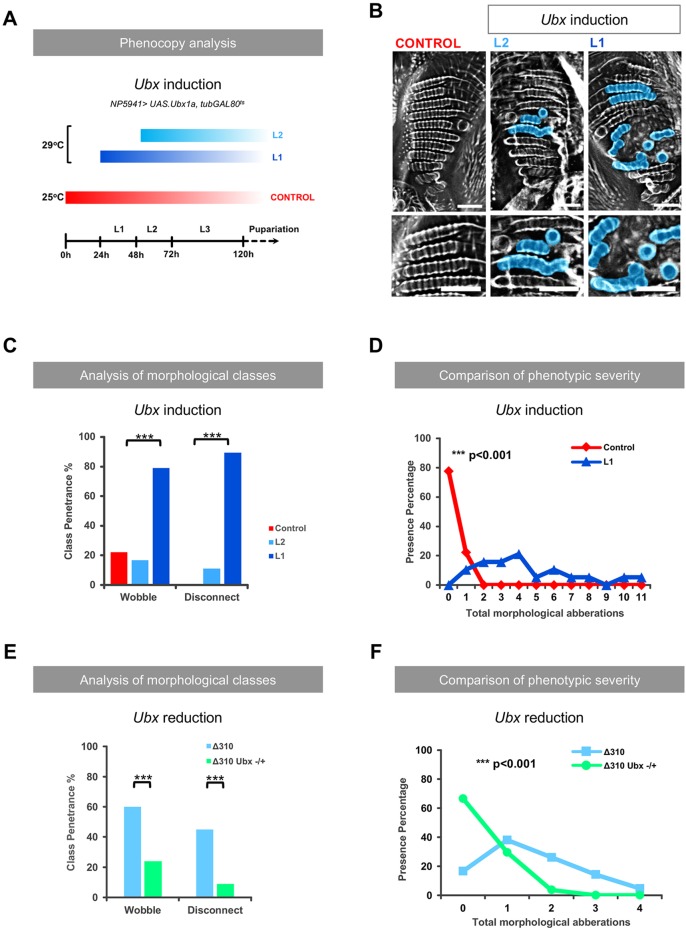
**The role of Ubx in miR-310C-dependent pedicel phenotypes.** (A) Experimental scheme used to increase *Ubx* expression within the transcriptional domain of miR-310C during different post-embryonic developmental stages. Induction of ectopic Ubx expression was achieved by shifting incubation temperature from 25°C to 29°C, which inactivates the temperature-sensitive GAL80 transcriptional repressor. (B) Comparison of pedicel phenotypes following induction of ectopic *Ubx* expression at first (L1) and second (L2) larval instar stages. Scale bars: 10 µm. (C) Phenotypic penetrance for wobble and disconnect morphological classes was calculated for each experimental condition. Statistical significance was determined using Fisher's exact test. (D) Total phenotypic severity was determined by quantifying all morphological aberrations per sample. Differences in distribution of control and L1 conditions was tested for statistical significance using Wilcoxon rank-sum test. *n*=18 (control), *n*=18 (L2), *n*=19 (L1). (E,F) Effect of Ubx reduction in miR-310C mutants. The *Δ310* deletion (w; *Δ310/Δ310)* was combined with a heterozygote loss-of-function allele of *Ubx^−/+^* (*w; ; Ubx ^abx, bx, pbx^/+*) to test the effects of Ubx reduction in a *Δ310* genetic background. (E) Morphological analysis of the pedicel showed a significant reduction in the penetrance of the wobble and disconnect phenotypes. (F) Analysis of the phenotypic severity of morphological aberrations within the pedicel shows a significant reduction when comparing the *Δ310 Ubx^−/+^* genotype (w; *Δ310/Δ310; Ubx ^abx, bx, pbx^/+*) with *Δ310*. Significance was tested using Fisher's exact test (C) and Wilcoxon rank-sum test (D), ***P<0.001. *n*=42 (*Δ310*)*, n*=54 (*Δ310 Ubx^−/+^*).

We then investigated whether a similar *Ubx*-independent mechanism was responsible for the patterning defects in pedicel sensilla and found that, in contrast, these anomalies were indeed dependent on *Ubx* expression. First, *Ubx* induction within the miR310C expression domain triggered at first larval stage (L1) does indeed lead to the generation of ‘disconnect’ and ‘wobble’ phenotypes at a statistically significant higher penetrance and severity than that observed in control flies (Fig. 5B-D; Fig. S2). Analysis of Ubx expression levels in conditions where Ubx expression was induced within the miR-310C transcriptional domain showed that there were subtle, but significant, increases in expression compared with wild-type discs, and that these levels were comparable with Ubx expression levels in Δ310 mutant discs. The effects of increased levels of Ubx expression are apparent only when induction of ectopic Ubx is initiated from L1 stage, suggesting the existence of a critical time period for setting Ubx expression levels with an impact on pedicel patterning. Previous work has indeed shown the existence of a critical period for the differentiation of haltere disc cells in relation to Ubx expression (Roch and Akam, 2000). We speculate that induction of Ubx post-L1 stage is not sufficient to achieve the levels of Ubx required to affect the phenotype studied here. Additionally, a reduction in Ubx expression in the *miR-310C* mutant context does indeed visibly reduce the effects of miRNA mutation (Fig. 5E-F) in line with the model that the control of Ubx expression level is a fundamental parameter for the development of anomalies in the patterning of haltere pedicel sensilla. All in all, these observations suggest that miRNA regulation controls Hox expression level during the development of fine-grain morphological patterning in *Drosophila*.

### The cellular basis for miR-310C effects on haltere patterning

**Fig. 6.**
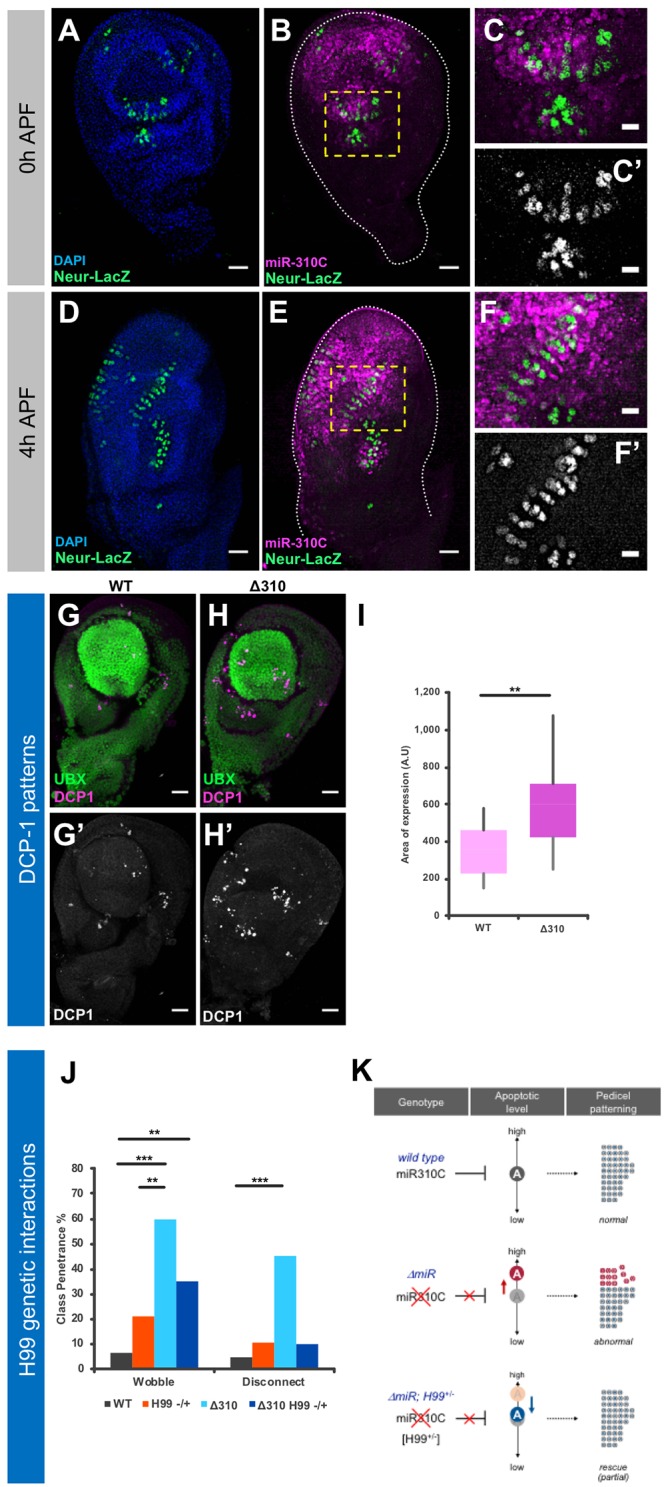
**Exploring the cellular basis underlying miR-310C effects on pedicel patterning.** (A-F′) To assess the relationship between developing sensory organ precursor cells (SOPs) and miR-310C-positive cells during haltere development, we used a reporter (*Neuralized-LacZ*) for the *neuralized* gene, a known marker for SOPs alongside *miR-310C::GAL4* to demark miR-310C domains of expression. (A-C′) Expression of *Neuralized-LacZ* (green) and *miR-310C* (magenta) in haltere discs at 0 h APF. SOPs are confined to specific regions of the disc corresponding to their future compartments. (B) Future dorsal and pedicel compartment SOPs are marked by a yellow rectangle. (C,C′) Enlarged dorsal pedicel and scabellum SOPs. (D-F′) Expression of *Neuralized-LacZ* and *miR-310C* in haltere disc at 4h APF. At this stage, the disc has begun to undergo an eversion process. (E) The dorsal pedicel SOPs (yellow box) have now formed an extended region containing rows of SOPs. (F,F′) Enlargements of dorsal pedicel SOPs. These precursors are surrounded by miR-310C-positive epithelial cells but do not express *miR-310C::GAL4* themselves. Scale bars: 25 μm in A,B,D,F; 10 μm in C,C′,F,F′. (G-I) Quantification of apoptosis in normal and miRNA mutant haltere discs. (G,H) Haltere imaginal discs co-stained for UBX (green) and DCP-1 (magenta), a marker for apoptotic cells. (G′,H′) Images showing only DCP-1 staining. Staining was performed in both wild-type and Δ310 (*w; Δ310/Δ310*) genotypes. Visual comparison shows an increase in apoptotic cells in Δ310 haltere discs. Scale bars: 10 μm in G-H’. (I) Quantification of DCP-1 expression within the imaginal discs. For each sample, the area of DCP-1 expression was calculated. The distribution of calculated areas was compared across genotypes. The size of the boxes represents the range of values that fit between the 25th and 75th percentiles of the distribution; the bottom and top whiskers represent the minimum and maximum values of the distribution, respectively. We detect a statistically significant increase in DCP1 expression in the Δ310 genotype (Student's *t*-test, **P<0.01). (J) Quantification of morphological class penetrance for wobble and disconnect phenotypes comparing wild-type, H99 (*w;; H99/TM6b*), Δ310 (*w; Δ310/Δ310*) and Δ310 H99^−/+^ (*w; Δ310/Δ310; H99/TM6b*) genotypes. Statistical significance was calculated using Fisher's exact test. **P<0.01, ***P<0.001. Data represent phenotypic penetrance. For apoptosis (I), *n*=15 (wild type) and *n*=*14* (*Δ310*)*.* For analysis of genetic interactions (J), *n*=44 (wild type), *n*=18 (*H99*), *n*=39 (*Δ310*), *n*=19 (*Δ310 H99^−/+^*). (K) Proposed model for the effects of miR-310C on pedicel patterning.

The nature of the wobble and disconnect phenotypes, where sensilla appear disorganised in different ways, suggests the possibility that the integrity of cells in the surrounding areas to the sensory precursors may be distorted by the miRNA mutation. To investigate this possibility, we sought to determine the spatial relationship between miR-310C ‘positive’ cells and sensory precursor cells during haltere disc development. To achieve this, we labelled miRNA expressing cells with miR-310>GFP and traced the sensory organ precursor (SOP) cells by means of *neuralized-lacZ* (Salzberg et al., 1997) at 0 and 4 h after puparium formation (AFP) (Fig. 6A-F). The results of these experiments show that sensory organ precursor (SOP) cells of the presumptive pedicel and scabellum are surrounded by miR-310C-positive cells (Fig. 6A-C) and that this spatial relationship is maintained during early pupal development (Fig. 6D-F). SOP precursors do not express *miR-310C::GAL4* themselves, in line with the phenotypic effects on the organisation (but not absence) of campaniform sensilla observed in the fully developed haltere (Fig. 4).

To advance our understanding of the actual cellular processes involved in the cellular rearrangement underlying the haltere phenotypes observed in miR-310C mutants, we considered the hypothesis that the absence of the miRNA may affect normal cell death patterns. To explore this possibility, we measured the influence of apoptotic processes in halteres derived from normal and miRNA mutant flies by quantifying the expression of the *Drosophila* effector caspase DCP-1 (Death caspase 1) (Song et al., 1997) and established that, in the absence of the miRNA, there is a statistically significant increase in apoptotic activity in the haltere disc (Fig. 6G-I). This supports to the notion that apoptosis is indeed dysregulated in miR-310C mutants. Furthermore, addition of a heterozygous mutation for the H99 deletion (White et al., 1994), which lacks the three pro-apoptotic genes *head-involution defective*, *grim* and *reaper* (*hid*, *grim* and *rpr*) (Abbott and Lengyel, 1991; Chen et al., 1996; White et al., 1994), to the miR-310C background compensates, at least to some degree, for the effects caused by the absence of miRNAs on the patterning of campaniform sensilla (Fig. 6J). In summary, these results suggest that apoptotic processes may play an influential role in the development of haltere sensilla and that absence of miR-310C activities may interfere with normal apoptotic patterns in the developing haltere.

## DISCUSSION

Our work investigates the roles of miRNA-dependent regulation of Hox expression during appendage development in *Drosophila*. The results presented above indicate that expression of *miR-310C* is complementary to that of Ubx within the haltere pouch and that overexpression of *miR-310C* in the haltere disc generates *Ubx*-like homeotic transformations in the resulting halteres. We also show that a fluorescent reporter bearing natural *Ubx* 3′UTR sequences is repressed when co-expressed with *miR-310C* during haltere formation and that deletion of *miR-310C* locus leads to an increase of *Ubx* expression in haltere discs and changes in haltere morphology. Based on these observations, we suggest that miRNA regulation of Hox expression contributes to the sculpting of fine-grain morphology in a *Drosophila* appendage.

Given the subtle nature of the changes in haltere morphology that emerge from miRNA-mediated Hox repression, it is legitimate to consider whether any such changes may be of any significance to the fly. Nonetheless, physiological and behavioural experiments strongly suggest that the fly is likely to care about the changes reported here. Classical work by Hugo Pflugstaedt (Pflugstaedt, 1912) and Fraenkel and Pringle (Fraenkel and Pringle, 1938) demonstrated that information transmitted from the mechanosensory sensilla at the base of the haltere is crucial for flight control. In particular, the campaniform sensilla on the dorsal side of the haltere sense cuticular stress during flight (Pringle, 1948; Wigglesworth, 1946) and this information influences wing commands. In this context, we predict that the rearrangements of sensory fields in the pedicel of *miR-310C* mutants are likely to jam the reading of stress and consequently send incorrect information to the centres responsible for flight control. Yet, at this point in time, it is difficult to design a clean test to tease apart the flight performance roles that emerge from Hox-dependent effects of *miR-310C* on campaniform sensilla from those caused by the noted Hox-independent change in haltere size.

Turning to the problem of the roles of miRNAs as modulators of morphogenetic processes, we envision that complex spatial distributions of specific gene repressors, such as miRNAs, may represent an important factor in the development of fine-grain structural patterns. According to this view, miRNAs may be agents that subdivide large developmental fields – primarily established through the action of transcription factors and signalling pathways – leading to the local modification of cuticular patterns so as to produce highly organised arrays of morphological elements. Such ‘micro-patterns’, many of which display remarkable reproducibility and functional relevance, include the campaniform sensilla treated here. miRNAs may thus play a very important role in the diversification of Hox inputs internal to the segment and provide a potential mechanistic explanation to the long-standing question of how individual developmental units such as insect segments (or vertebrate rhombomeres) can decompose an axially arranged Hox-code into the remarkable spectrum of cell differentiation programmes that produce features within the segment. In this scenario, we propose an interesting new link between the emergence of Hox-dependent fine-grain morphology and three molecular processes concerning the expression and function of miRNAs. These include: (1) the transcriptional control of miRNA precursors so that miRNA genes achieve an adequate expression level at the right spatio-temporal coordinates within the developing organism; (2) the control of miRNA biogenesis (which requires a whole panoply of factors) so as to ensure the release of a functional miRNA molecule in sufficient quantity to exert biological effects; and (3) the strength and specificity of interaction of miRNAs with particular Hox mRNA elements within specific cellular environments; this latter element is deemed to be under the indirect influence of other potentially competing targets co-expressed in such locations. If our proposal is correct, genetic variation affecting all these three processes may ultimately be related to the emergence of Hox-dependent patterning at the microscale level.

How may the activities of miR-310C and *Ubx* modify the cellular processes that underlie the formation of complex patterns of campaniform sensilla in the pedicel? So far, this remains an unanswered question. Given the nature of the wobble and disconnect phenotypes it may be plausible that when *Ubx* is expressed at higher levels than normal, new cells end up inserted in between the individual sensilla. In this context, our analysis of caspase expression and genetic interaction experiments suggests that apoptosis could play an active role in pruning the excess of cells during normal haltere formation. We speculate that an adequate level of apoptosis must occur during haltere development to produce normal pedicel patterning (Fig. 6K). Removal of miR-310C leads to increased levels of apoptosis and a concomitant abnormal pattern of the pedicel, and an attenuation of apoptotic activities in miRNA mutants presumably compensates apoptotic levels and alleviates the generation of patterning effects.

It is also possible that a surge in Ubx expression may disrupt normal apoptosis. Hox genes can regulate apoptosis during embryogenesis (Lohmann et al., 2002) so it is conceivable that disruption of an apoptotic-inducing function by means of miRNA-dependent increased levels of *Ubx* could potentially lead to abnormal incorporation of cells to the haltere sensory field, displacing and re-arranging sensilla as seen in our results.

Our work shows that miRNA regulation can influence the expression of Hox genes and contribute to the emergence of morphological patterns in the *Drosophila* haltere. Based on our results, and on the evolutionary conservation of the Hox system (Mallo and Alonso, 2013) and that of the *miR-310C* system – which belongs to an evolutionary ancient family of miRNAs (Ninova et al., 2014) – we propose that the relationship between miRNAs and Hox genes may also influence the development of fine-grain morphology in other bilaterian organisms.

## MATERIALS AND METHODS

### *Drosophila* strains

*Drosophila* strains were cultured following standard procedures at 25°C. Oregon Red was used as a wild-type strain. The following *Ubx* alleles were used: *bx^34e^*, *Ubx^1^* and *Ubx ^61d^ pbx^1^* composite chromosome (Bloomington, #3437, #529, #3425). The *Δ310* deletion was created via P-element excision of the *miR-310C^EP2587^* insertion (Tang et al., 2010). For genetic interactions with Ubx, the *Δ310* allele was crossed into a Ubx-deficient background using *abx^1^ bx^3^ pbx^1^* mutant chromosome. The following GAL4 and UAS stocks were used: *miR-310C^NP5941^*GAL4 (Bloomington, #113798), *nubbin::GAL4* (Bloomington, #38418), *UbxGAL4^LDN^* (de Navas et al., 2011), *UAS::mCh-K10* and *UAS::mCh-Ubx-3′UTR* (Thomsen et al., 2010), *w;;UAS::Ubx1a, tubGAL80^ts^* (Pavlopoulos and Akam, 2011; Reed et al., 2010) and *UAS::nls-mCherry, UAS::nls-GFP.* Clone induction was achieved using *w hsFLP; UAS::myrRFP/Cyo; Act.FRT.STOP.GAL4/TM6b* (a gift from Rob Ray, HHMI Janelia Research Campus, USA). To identify developing SOP cells, we used a known SOP marker, *Neuralized-LacZ* transgene (Bloomington, #4369). For analysis of the apoptosis pathway, we used the *Df(3L)H99* allele (Bloomington, #1576). For genetic interactions between the apoptotic pathway and *miR-310C*, the *miR-310C Δ310* allele was crossed into the *Df(3L)H99* background.

### PITA analysis and sequence alignment

miRNA target predictions were carried out using the PITA algorithm (Kertesz et al., 2007) (genie.weizmann.ac.il/pubs/mir07/mir07_prediction.html). The *Ubx* extended 3′UTR sequence used for the target predictions was extracted from FlyBase (flybase.org). Results were filtered to include only miRNAs that were detected in imaginal discs using next-generation RNA-sequencing (Ruby et al., 2007). *miR-310C* pre-miRNA sequences were extracted from FlyBase and aligned using the ClustalW tool from EBI (www.ebi.ac.uk/Tools/msa/clustalw2/)

### Adult cuticle preparations

Haltere appendages were analysed in detail following cuticle dissection and preparation as described previously (de Navas et al., 2006). Samples were stored in an ethanol/glycerol (3:1) mixture. Flies were macerated in 10% KOH at 60°C for 60 min. Samples were washed and stored in ethanol/glycerol (3:1) before mounting in 70% glycerol/PBT solution.

### RNA-Seq data analysis

Full details will be published elsewhere (R.K., A. D. Bomtorin, Z. L. Simões and C.R.A., unpublished). Briefly, total RNA was extracted from isolated wing and haltere imaginal discs. Small RNA was extracted and cDNA libraries generated using Illumina TruSeq Small RNA sample preparation kits (Illumina). Sequencing data underwent quality control and filtering using the GALAXY platform (https://usegalaxy.org/). Filtered reads were aligned to the genome using Bowtie tool (Langmead et al., 2009) and quantified using Cufflinks (Trapnell et al., 2010). Analysis of data was performed using Microsoft Excel and R Statistical Computing (www.r-project.org/).

### Immunohistochemistry

Imaginal discs were fixed following dissection in 4% paraformaldehyde and stored at −20°C in 100% methanol. Immunostaining were based on standard protocols (Nagaso et al., 2001). Briefly, samples were rehydrated from 100% methanol to 1×PBS solution. Samples were pre-treated with 80% acetone at −20°C for 20 min, washed, then re-fixed with 4% paraformaldehyde and finally washed in 1×PBTwx (1×PBS, 0.1%Tween20, 0.1%TritonX). Samples were then blocked with 1% milk solution (Milkpowder, PBT) for 30 min. Primary antibodies were incubated overnight at 4°C and then washed in PBTwx and secondary antibodies incubated for 2 h at room temperature. Samples were then washed in PBTwx and stored in 70% glycerol/PBTwx at 4°C to await analysis. Primary antibodies used were mouse anti-Ubx FP3.38 (1:20; a gift from Rob White, University of Cambridge, UK), rabbit anti-RFP (Invitrogen, 1:1000), rat anti-RFP (Chromotek, 1:1000), rabbit anti-Betagalactosidase (Promega, 1:300), rabbit anti-GFP (Molecular Probes, 1:1000) and rabbit anti-DCP-1 (Cell Signalling Technologies, 1:200). Secondary antibodies used were anti-mouse Alexa A488 (Invitrogen, 1:300), anti-rabbit FITC (Jackson Immunoresearch, 1:300), anti-rat Cy3 (Jackson Immunoresearch, 1:300), and anti-mouse A647 (Jackson Immunoresearch, 1:300).

### mCherry expression analysis

To test the ability of *miR-310C* miRNAs to target the *Ubx*-3′UTR, *UAS::mCh-UbxΔPAS* was co-expressed with either *UAS::miR310* or *UAS::nls-*GFP using *UbxGAL4^LDN^*, a GAL4 driver known to be active in the developing haltere imaginal disc. Haltere discs were dissected from developmentally staged white pre-pupae, stained for RFP, GFP and Ubx (see above), and imaged for further downstream analysis.

### RT-PCR/genomic PCR

Total RNA was extracted from all samples using Tri-Reagent (Ambion) following the manufacturer's standard protocol. cDNA synthesis from total RNA was carried out using RETROscript Kit (Ambion) using the standard manufacturer's protocol. For each synthesis reaction, 1 µg RNA was treated with DNAseI (New England Biolabs) before cDNA synthesis was carried out. Expression values were normalised using *Rpl32 (Rp49)*. Primer pairs used were as follows: *Ubx Universal*, 5′-AGTGGAAGGAGCGCAGATTA-3′ and 5′-CAGAATTTTGCTCGCATTCA-3′; *Ubx Distal*, 5′-GAACGAAGGC AGATGCAAAT-3′ and 5′-GGTAAGTGGTCGGATGCAGT-3′; and *rp49*, 5′-CCAGTCGGATCGATATGCTAA-3′ and 5′-TCTGCATGAGCAGGACCTC-3′. To validate the *Δ310* genotype, the following primer pairs were used: *Quasimodo*, 5′-TTCGGTGTGGTTTCGAGTCT-3′ and 5′-GCAAACACACACAGCGAGTT-3′; *Nnf1a*, 5′-TGCTATGGCCAAGAGCAAT-3′ and 5′-TTGTCAGAAGTCGTTCAATGC-3′; *Black*, 5′-GACAGGGTGATACGCCATTT-3′ and 5′-AGACTTTGATGCCACCGAAC-3′; *miR-310*, 5′-CCGGCCTGAAAATATCAAGA-3′ and 5′-GAGAAAAGCGAACTGGATT-3′; and *miR-313* 5′-TACCCGACATCGTCTAGCC -3′ and 5′ AAAATGCAGAATTGCCCTTG-3′.

### Expression quantification and image analysis

All data analysis was performed using Microsoft Excel and R statistical computing. Expression intensity of mCherry constructs and Ubx was determined using the measure function in Fiji (fiji.sc/). Ubx expression intensity patterns within the haltere of wild-type and *Δ310* genotypes were measured using the Plot Profile tool in Fiji. For both analyses, statistical significance was determined using Student's *t*-test. Haltere capitellum size was determined using the measure function in Fiji and statistical significance was determined using Student's *t*-test. Analysis of morphological changes within the haltere pedicel was achieved by imaging each haltere sample and manually documenting any morphological aberrations. Statistical significance of morphological penetrance was determined using Fisher's exact test. Statistical significance of overall phenotypic severity was determined using the Wilcoxon rank-sum test. For all statistical testing: *P<0.05, **P<0.01, ***P<0.001.

### Timed induction of Ubx overexpression

The GAL4/UAS/GAL80 system was used to control the timing of induction of Ubx overexpression within the developing haltere. Briefly, the *w;;UAS::Ubx1a, tubGAL80^ts^* and *miR-310C^NP5941^GAL4* stocks were crossed and maintained at 18°C. At appropriate developmental times, progeny of the cross were temperature shifted to 29°C, inducing the activity of the *UAS::Ubx1a* transgene.

### Imaging and microscopy

Samples for scanning electron microscopy were prepared using the following protocol. Adult flies were dehydrated in 25%, 50% and 100% ethanol solutions. Instead of critical point drying, samples were washed three times with hexamethyldisilazane (Sigma-Aldrich). The solvent was left to evaporate by air drying the samples for 24 h. The adult thorax with the haltere appendages attached was isolated from the rest of the animal to allow for better manipulation when mounting samples. Fluorescence microscopy was performed using either a Leica DFC6000 or Leica SP*5* microscopes. For detailed analysis of haltere sensory structures, samples were illuminated with UV light, resulting in significant auto-fluorescence, and imaged using Leica DFC6000.

## Supplementary Material

Supplementary information
