# Supplementary material for: MicroRNA-dependent regulation of Hox gene expression sculpts fine-grain morphological patterns in a Drosophila appendage
Source: Development. 2018 Oct 16;145(20):dev161133. doi: 10.1242/dev.161133 (PMC6215401; doi:10.1242/dev.161133)
Supplement: Supplementary information [file develop-145-161133-s1.pdf]

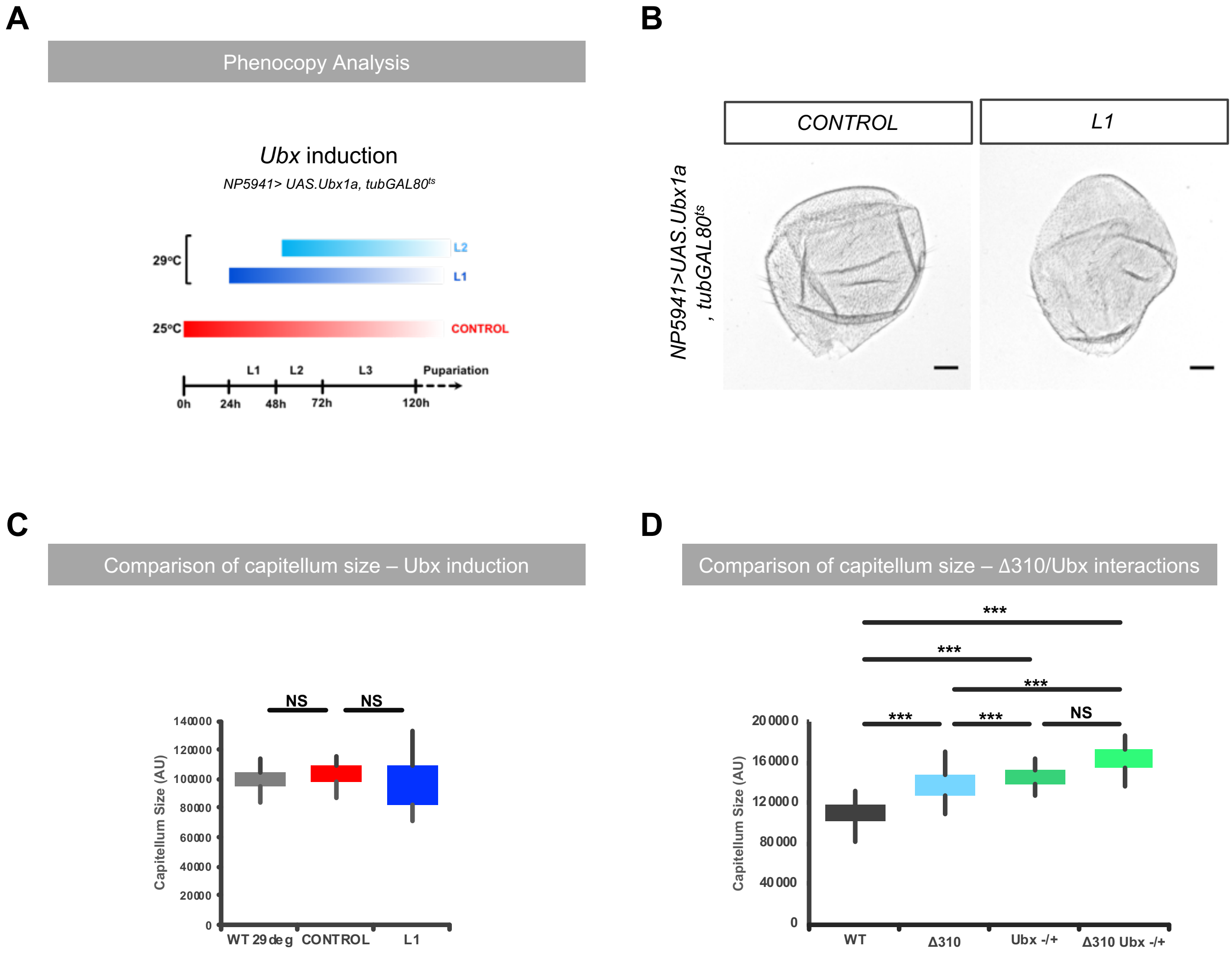

Figure S1. Effects of Ubx induction on capitellum size. (A) Representation of the experimental scheme used to express *Ubx* during post-embryonic development within the *miR310C::GAL4* expression domain. (B) Sample images of capitella from Control and L1 test groups. (C) Quantification of *capitellum* size in halteres from wild-type strains housed at 29ºC (WT 29deg; grey), Control (red) and L1-induced (blue) groups. No significant differences were detected among the genotypes. (D) To determine if the effects of the Δ310 allele on capitellum size were due to increased expression of Ubx, we quantified capitellum size in halteres lacking miR-310C in a *Ubx* deficient genetic background (*w ; Δ310/Δ310 ; abx^1 bx^3 pbx^1/+*). Results show that addition of a *Ubx* loss-of-function allele leads to a further increase in capitellum size demonstrating that a reduction of *Ubx* expression does not “rescue” the Δ310 phenotype and instead magnifies the miRNA mutant phenotype. Differences in size distributions were tested for statistical significance using Students t-test, *** $p< 0.001$.

Development • Supplementary information

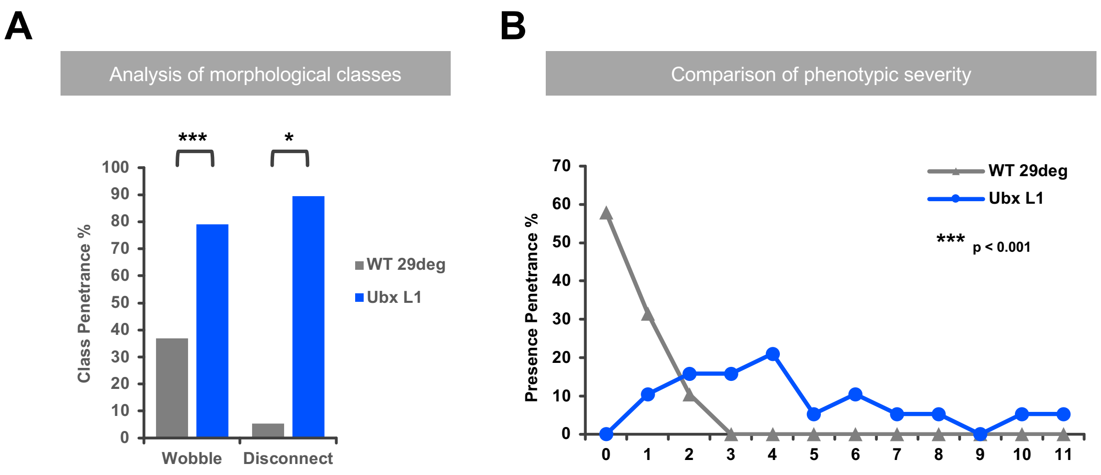

Figure S2. Effect of high temperature on the formation of haltere sensory cells. Wild-type halteres from flies housed at 29°C (WT 29deg) were compared to Ubx L1 'gain-of-function' (Ubx L1) halteres expressing Ubx within the miR-310C domain. (A) Analysis of morphological changes shows that both Wobble and Disconnect phenotypic classes were significantly enriched in Ubx L1 halteres (Fishers Exact test, * $p<0.05$, *** $p<0.001$). Comparison of phenotype severity also shows that Ubx L1 halteres are significantly enriched for morphological aberrations (Wilcoxon rank-sum test, *** $p<0.001$) [n numbers were: WT 29deg=19, Ubx L1=19].
